# Chaperones Ameliorate Beta Cell Dysfunction Associated with Human Islet Amyloid Polypeptide Overexpression

**DOI:** 10.1371/journal.pone.0101797

**Published:** 2014-07-10

**Authors:** Lisa Cadavez, Joel Montane, Gema Alcarraz-Vizán, Montse Visa, Laia Vidal-Fàbrega, Joan-Marc Servitja, Anna Novials

**Affiliations:** 1 Diabetes and Obesity Research Laboratory, Institut d'Investigacions Biomèdiques August Pi i Sunyer (IDIBAPS), Hospital Clínic de Barcelona, Spain; 2 Centro de Investigación Biomédica en Red de Diabetes y Enfermedades Metabólicas Asociadas (CIBERDEM), Barcelona, Spain; Universidad Miguel Hernández de Elche, Spain

## Abstract

In type 2 diabetes, beta-cell dysfunction is thought to be due to several causes, one being the formation of toxic protein aggregates called islet amyloid, formed by accumulations of misfolded human islet amyloid polypeptide (hIAPP). The process of hIAPP misfolding and aggregation is one of the factors that may activate the unfolded protein response (UPR), perturbing endoplasmic reticulum (ER) homeostasis. Molecular chaperones have been described to be important in regulating ER response to ER stress. In the present work, we evaluate the role of chaperones in a stressed cellular model of hIAPP overexpression. A rat pancreatic beta-cell line expressing hIAPP exposed to thapsigargin or treated with high glucose and palmitic acid, both of which are known ER stress inducers, showed an increase in ER stress genes when compared to INS1E cells expressing rat IAPP or INS1E control cells. Treatment with molecular chaperone glucose-regulated protein 78 kDa (GRP78, also known as BiP) or protein disulfite isomerase (PDI), and chemical chaperones taurine-conjugated ursodeoxycholic acid (TUDCA) or 4-phenylbutyrate (PBA), alleviated ER stress and increased insulin secretion in hIAPP-expressing cells. Our results suggest that the overexpression of hIAPP induces a stronger response of ER stress markers. Moreover, endogenous and chemical chaperones are able to ameliorate induced ER stress and increase insulin secretion, suggesting that improving chaperone capacity can play an important role in improving beta-cell function in type 2 diabetes.

## Introduction

One of the major pathological features of the pancreas in type 2 diabetes (T2D) is the presence of islet amyloid deposits, found in more than 80% of patients at autopsy [1]. These deposits are implicated in the process of β-cell deterioration and reduction in beta-cell mass and involve islet amyloid polypeptide (IAPP) aggregation of monomers into oligomers, fibrils and, ultimately, mature amyloid deposits [2], [3], [4], [5].

The endoplasmic reticulum (ER) is the site of several important functions, including the synthesis, folding and maturation of secreted proteins. The pancreatic beta-cell has an extremely developed ER enabling the secretion of proteins such as insulin or IAPP [6], [7]. However, the accumulation of misfolded proteins can alter ER homeostasis [7]. As a consequence, cells activate a succession of signal transduction cascades termed unfolded protein response (UPR), which may trigger inflammation and, ultimately, cell death [7], [8], [9], [10].

To address the problem of misfolded proteins, cells have developed complex mechanisms that assist correct folding in the ER. Folding factors termed chaperones bind unfolded secretory proteins and prevent them from misfolding and aggregating [11]. Previous studies have shown that beta-cell-specific overexpression of glucose-regulated protein 78 kDa (GRP78, also known as BiP) can protect transgenic mice from disturbances in ER homeostasis, such as glucose intolerance and insulin resistance, induced by high-fat diet treatment [12]. In addition, adenoviral BiP overexpression is able to reduce ER stress [13] and reverse hyperglycemia- and hyperlipidemia- induced insulin synthesis and secretion *in vitro* [14]. Similarly, protein disulfide isomerase (PDI), which catalyzes the formation and breakage of disulfide bonds, has been shown to have important benefits as a chaperone [15], [16] and to play a key role in the ER-associated protein degradation process [17]. Recent reports indicate beneficial effects of chemical or pharmacological chaperones, such as taurine-conjugated ursodeoxycholic acid (TUDCA) or 4-phenylbutyrate (PBA), in relieving ER stress and improving protein folding [18], [19], [20], particularly through oral treatment in rodent models of obesity and T2D [18]. Moreover, increasing evidence in neurodegenerative disorders points to a role of chaperones in preventing the protein aggregation leading to attenuation in ER stress [21], [22], [23]. Since this is a common feature also observed in T2D, accumulation of misfolded human IAPP (hIAPP) may be responsible for the progression of the disease.

Our group and others have previously demonstrated that extracellular hIAPP aggregation is associated with ER stress responses in mouse beta-cells [24], [25]. Furthermore, we have detected toxic intracellular aggregates in a rat pancreatic beta-cell line overexpressing hIAPP, which lead to a defective insulin and IAPP secretion in response to glucose [26]. Nevertheless, the role of hIAPP overexpression and ER stress induction has not been fully clarified.

The main objective of this work is to elucidate the role of endogenously produced hIAPP in ER stress induction in a rat pancreatic beta-cell line. In addition, we aim to study whether chaperones can ameliorate exogenously-induced ER stress in order to identify new targets for preventing the loss of beta-cell function in T2D. In the present study, we show that hIAPP potentiates induced ER stress in hIAPP-expressing beta cells. Moreover, the overexpression of molecular chaperones, or treatment with chemical chaperones, is able to ameliorate induced-ER stress and restore insulin secretion. Our results suggest that improving chaperone capacity may be important for diminishing stress in hIAPP-expressing cells, which ultimately can impact the formation of amyloid deposits in T2D.

## Materials and Methods

### Cell culture and treatments

A rat pancreatic beta-cell line INS1E overexpressing human and rat IAPP was previously established by our laboratory [26]. Briefly, these cells were stably transfected with hIAPP cDNA (hIAPP-INS1E cells), rat IAPP (rIAPP-INS1E) or an empty vector (INS1E control) under the cytomegalovirus promoter (CMV). Cells were maintained in complete RPMI 1640 (Sigma) supplemented with 1 mM sodium pyruvate (Thermo Scientific), 10 mM HEPES (Thermo Scientific), 10% Fetal Bovine Serum, 2 mM L-glutamine, 5 µM beta-mercaptoethanol, 200 µg/ml geneticin (Gibco) and 100 units/ml of penicillin and 100 µg/ml streptomycin at 37°C with 5% CO_2_. Palmitic acid (400 µM, Sigma), combined with 25 mM of glucose, was added to the cells by conjugating with 1% (w/v) of albumin from bovine serum (BSA, Sigma). Thapsigargin (Sigma) was diluted in DMSO and used at a final concentration of 0.5 µM. PBA (Sigma) was dissolved in PBS and used at 2.5 mM. TUDCA (Calbiochem) was diluted in water and used at 200 µM.

### Adenoviral transduction

The recombinant adenoviruses encoding for BiP and PDI/GFP under the CMV promoter were produced following earlier protocols [14], [27]. hIAPP-, rIAPP- and control INS1E cells were transduced with 20 MOI of Ad/CMV-BiP, Ad/CMV-PDI or Ad/CMV-GFP, and incubated at 37°C and 5% CO2 for 2 hours. Cells were then washed with PBS and incubated at 37°C and 5% CO2 for 24 hours in fresh media RPMI 1640 prior to further treatment.

### Cell viability assay

LIVE/DEAD Viability/Cytotoxicity Kit (Invitrogen) was performed following the manufacturer's instructions. Briefly, adenoviral transduced cells were cultured in the presence of 20 MOI of Ad-BiP. Cells were washed with PBS, and combined LIVE/DEAD assay reagents were applied for 30 min at room temperature. Images were captured using an Olympus Bx61 microscope and In Vivo or DP controller software. Thapsigargin treatment for 24 hours was used as positive control.

### Small interfering RNA transfection

Knockdown of CCAAT/enhancer-binding protein (C/EBP) homologous protein (CHOP) expression in cells was performed using 20 µM of small interfering RNA (Invitrogen) or 20 µM of scramble siRNA (Applied Biosystems) as a control using Metafecten Pro (Biontex), according to the manufacturer's instructions. At six hours, the medium was changed to complete RPMI. Twenty-four hours after transfection, cells were treated with either, thapsigargin or 25 mM glucose and BSA-coupled palmitic acid (400 µM) for another 24 hours.

### RNA isolation and Real-Time qPCR

Total RNA from cells were extracted using TRIZOL reagent (Invitrogen) following the manufacturer's instructions. Reverse transcription was performed with 0.5–1 µg of total RNA using Superscript III (Invitrogen), following the manufacturer's instructions. Real-Time PCR (RT-PCR) was carried in duplicates with 2 µg of transcribed cDNA and MESA Green qPCR MasterMix Plus FOR SYBR (Eurogentec) in a LightCycler 480 II sequence detection system (Roche Applied Science). PCR products were verified through dissociation curve analysis using SDS software (Roche Applied Science). Expression levels were normalized to TATA box-binding protein 1 (Tbp1) mRNA and represented in arbitrary units. The sets of primers used in these experiments can be seen in Table 1.

**Table 1:** List of primer sequences used in qRT-PCR experiments.

Primer	(5′----3′)	Species	Gene ID
ATF3 Forward	GCTGGAGTCAGTCACCATCA	Rat	Atf3
ATF3 Reverse	ACACTTGGCAGCAGCAA	Rat	Atf3
CHOP Forward	CCAGCAGAGGTCACAAGCAC	Rat	Ddit3
CHOP Reverse	CGCACTGACCACTCTGTTTC	Rat	Ddit3
*Spliced* XBP1 Forward	GAGTCCGCAGCAGGTG	Rat	Xbp1
*Spliced* XBP1 Reverse	GCGTCAGAATCCATCCATGGGA	Rat	Xbp1
TBP1 Forward	GAGATCACCCTGCAGCATCA	Rat	Tbp
TBP1 Reverse	GCAGTGCCGCCCAAGTAG	Rat	Tbp
BiP/GRP78 Forward	TGCAGCAGGACATCAAGTTC	Rat	Hspa5
BiP/GRP78 Reverse	AAAGAAGACCCCGTTTACAG	Rat	Hspa5
ATF3 Forward	TCGGATGTCCTCTGCGCTGGA	Mouse	Atf3
ATF3 Reverse	CTGACTCTTTCTGCAGGCACTCTGT	Mouse	Atf3
CHOP Forward	AAGATGAGCGGGTGGCAGCG	Mouse	Ddit3
CHOP Reverse	GCACGTGGACCAGGTTCTGCT	Mouse	Ddit3
*Spliced* XBP1 Forward	GAACCAGGAGTTAAGAACACG	Mouse	Xbp1
*Spliced* XBP1 Reverse	AGGCAACAGTGTCAGAGTCC	Mouse	Xbp1
TBP1 Forward	ACCCTTCACCAATGACTCCTATG	Mouse	Tbp
TBP1 Reverse	ATGATGACTGCAGCAAATCGC	Mouse	Tbp
BiP/GRP78 Forward	TGCAGCAGGACATCAAGTTC	Mouse	Hspa5
BiP/GRP78 Reverse	TACGCCTCAGCAGTCTCCTT	Mouse	Hspa5

### Western blot analysis

Cultured cells were washed in PBS and lysed in an ice-cold lysis buffer (50 mmol/l Tris Ph 7.5, 5 mmol/l EDTA, 150 mmol/NaCl, 1% Triton X-100, 10 mmol/l sodium phosphate, 10 mmol/l sodium fluoride, 10 mmol/l and 10% of proteases inhibitors) for 20 min on ice, followed by centrifugation at 12′000×g for 20 min at 4°C. Protein concentration in the supernatant was determined using Bio-Rad Protein Assay kits (Bio-Rad), following the manufacturer's instructions. Protein samples (20–30 µg) were resolved by SDS-PAGE and transferred to PVDF membranes (PerkinElmer Life Sciences). Membranes were blocked for 2 hours with 5% skim milk or 5% BSA and incubated overnight at 4°C in primary antibodies: BiP/GRP78 (1∶1000, Santa Cruz), peIF2α (1∶1000, Cell Signaling), ATF3 (1∶1000, Santa Cruz), CHOP (1∶1000, Cell Signaling) and anti-β-actin antibody (1∶1000). Membranes were washed with TBST and incubated with horseradish peroxidase-conjugated secondary antibodies (GE Healthcare) for 2 hours. Immunoreactive protein bands were developed with an ECL chemiluminescence reagents kit (Pierce). Changes in protein levels were evaluated by Quantity One software (Bio-Rad laboratories).

### Glucose Stimulated Insulin Secretion (GSIS) Assay

hIAPP-INS1E cells were transduced overnight with 20 MOI of Ad-GFP, Ad-BiP, Ad-PDI, TUDCA or PBA. Cells were preincubated in a Krebs-Ringer bicarbonate buffer (KRB) containing 140 mM NaCl, 4.5 mM KCl, 2.5 mM CaCl2, 1 mM MgCl2.6H2O, 20 mM HEPES, pH 7.4, and supplemented with 0.1% BSA and 2.8 mM glucose for 30 min at 37°C with 5% CO2, following by stimulation with 2.8 mM or 16.7 mM glucose KRB for 1 hour at 37°C. Supernatant was recovered and cells were lysed in 500 µl of acid-ethanol solution to measure insulin content. Insulin levels and contents were determined by insulin ELISA (Mercodia), according to the manufacturer's protocol.

### Immunohistochemistry

hIAPP-INS1E cells were fixed in 4% paraformaldehyde for 10 minutes. After blocking with PBS in 0.2% FBS cells for 1 hour, cells were immunostained using guinea pig anti-insulin (Dako) and rabbit anti-caspase 3 antibodies (Cell signaling), and goat anti-guinea pig Alexa Fluor 594 and goat anti-rabbit Alexa Fluor 594 (Molecular Probes) as secondary antibodies.

### Statistical analysis

Statistical analysis between two groups was performed using Student's two-tailed t test and differences among more than two groups were carried out by ANOVA followed by Tukey test. Differences were considered significant when *p<0.05. Data in bar graphs are represented as mean ± SEM.

## Results

### ER stress-induced apoptosis in hIAPP-INS1E cells

**Figure 1:**
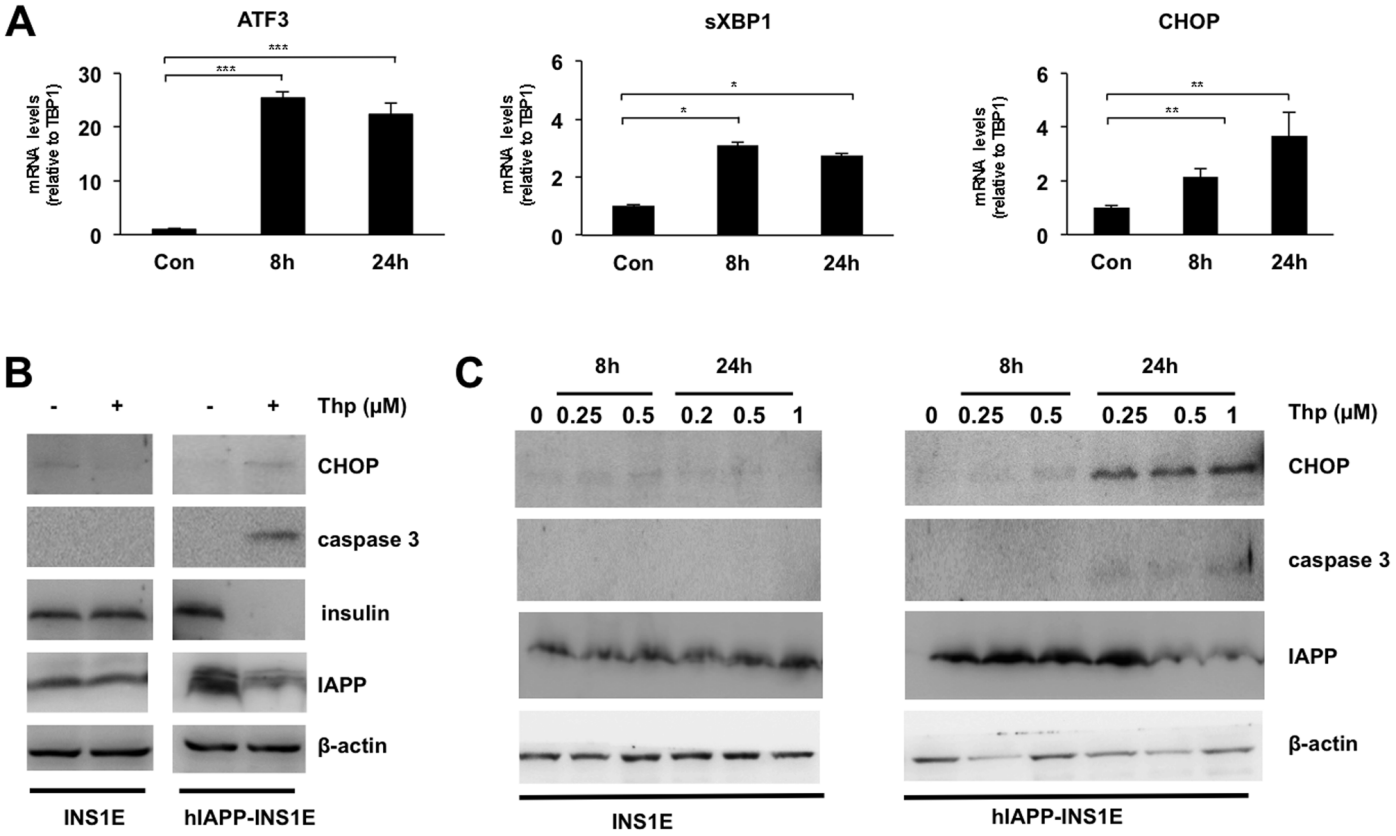
Thapsigargin induces ER stress and apoptosis in hIAPP-INS1E cells. A) INS1E control cells were cultured at 11 mM glucose and treated with 1 µM of thapsigargin for 8 and 24 hours. mRNA levels of ER stress markers ATF3, sXBP1 and CHOP were quantified by Real-Time PCR. *p<0.05, **p<0.01 and ***p<0.001 versus control cells at time 0. Results are expressed s mean ± S.E.M from five independent experiments. B) INS1E and hIAPP cells were exposed to 1 µM thapsigargin for 24 hours. Expression levels of CHOP, cleaved caspase 3, insulin, IAPP and β-actin were determined by Western blot. C) INS1E and hIAPP-INS1E control cells were exposed to 0.25, 0.5 and 1 µM of thapsigargin (Thp) for 8 and 24 hours. Protein levels of CHOP, cleaved caspase 3, IAPP and β-actin were determined by Western blot (n = 3). Representative Western blotting images are shown from 2 to 3 independent experiments.

To examine whether hIAPP overexpression potentiates ER stress in beta-cells, our group established a model in which a rat pancreatic beta-cell line, INS1E, was stably transfected with either hIAPP (hIAPP-INS1E cells), rIAPP (rIAPP-INS1E) or an empty vector (INS1E control cells) [26]. After treatment with chemical ER stress inducer thapsigargin at 1 µM for 8 and 24 hours, INS1E cells increased expression levels of ER stress genes such as activating transcription factor 3 (ATF3), spliced X-box binding protein 1 (sXPB1) and CCAAT/enhancer-binding protein (C/EBP) homologous protein (CHOP) (Figure 1A). Furthermore, 1 µM thapsigargin treatment for 24 hours did not increase the protein expression of CHOP or cleaved caspase 3 and did not affect the expression levels of insulin or IAPP (Figure 1B, left panel). However, treatment of hIAPP-INS1E cells with 1 µM of thapsigargin for 8 hours induced ER stress and severe apoptosis, by activation of CHOP and cleaved caspase 3 (Figure 1B, right panel). In addition, thapsigargin treatment decreased insulin and hIAPP production (Figure 1B, right panel), indicating that 1 µM of thapsigargin was associated with high toxicity in hIAPP-INS1E cells. Thus, to determine the optimal thapsigargin doses in hIAPP-expressing cells, we performed a dose/response experiment (0.25, 0.5 and 1 µM) at 8 and 24 hours. hIAPP-INS1E cells showed a high activation of ER stress marker CHOP and effector cleaved caspase 3 at 24 hours (Figure 1C). Furthermore, hIAPP-INS1E cells lost hIAPP expression, suggesting that either, 0.5 or 1 µM thapsigargin at 24 hours proved to be lethal in these cells (Figure 1C). Nevertheless, the dose of 0.5 µM of thapsigargin at 8 hours showed a mild activation of CHOP, absence of cleaved caspase 3 and unaffected hIAPP expression. Thus, a dose of 0.5 µM for 8 hours was chosen for further experiments based on mild ER stress induction in the absence of apoptosis.

### hIAPP-INS1E cells are more sensitive to ER stress inducers

**Figure 2:**
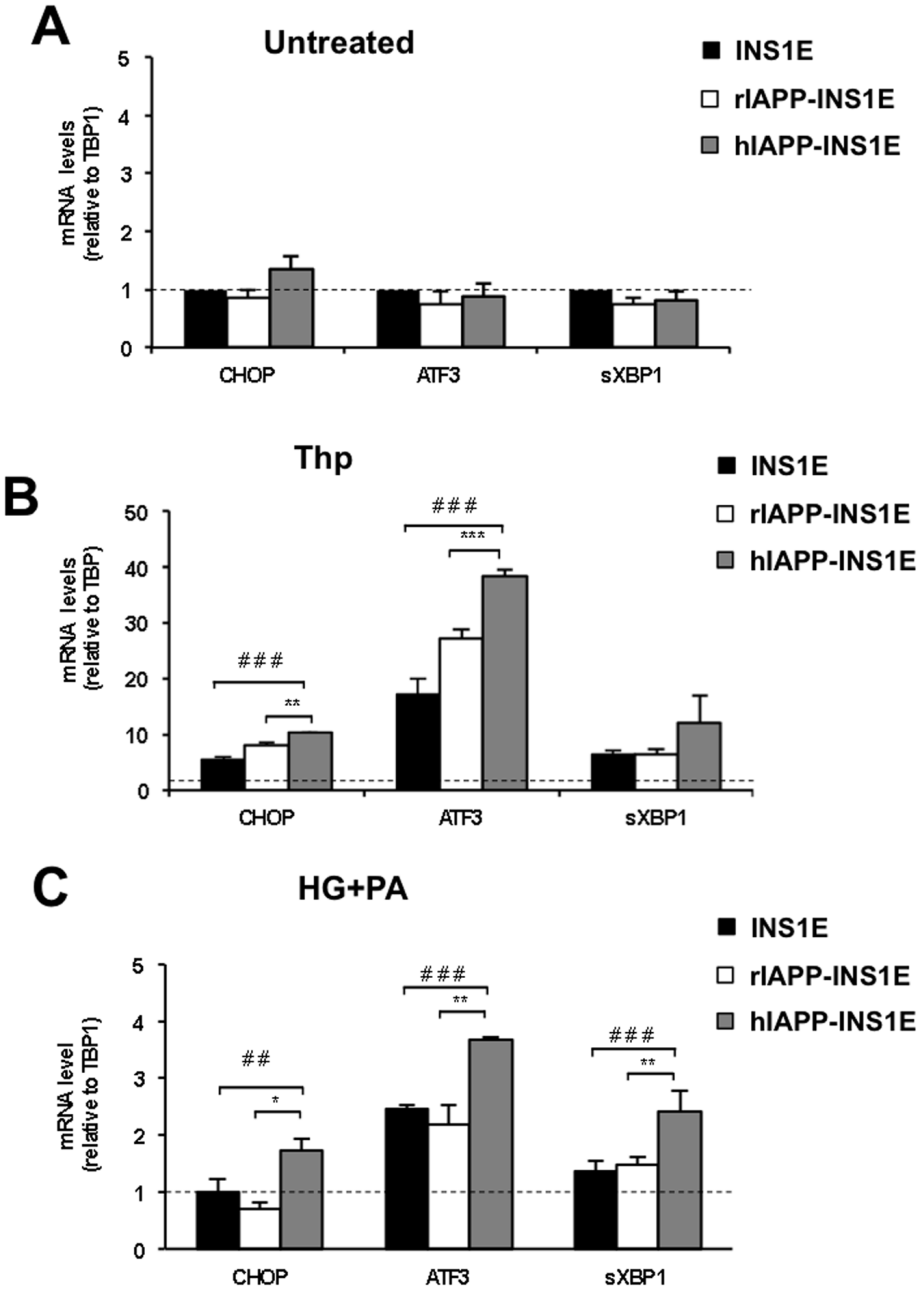
Thapsigargin and high glucose and palmitic acid potentiate ER stress gene expression in hIAPP-INS1E cells. ER stress expression markers CHOP, ATF3 and sXBP1 were determined by real-time PCR from hIAPP-INS1E, rIAPP-INS1E and INS1E control cells cultured at A) 11 mM glucose B) 11 mM glucose exposed to 0.5 µM of thapsigargin for 8 hours or C) 25 mM of glucose (HG) with 400 µM palmitic acid (PA) for 24 hours. Results are normalized to untreated INS1E, rIAPP-INS1E or hIAPP-INS1E cells (dashed line) and expressed as mean ± S.E.M from five independent experiments. *p<0.05 **p<0.01 and ***p<0.001 *vs*. rIAPP and *^##^p*<0.01, *^###^p*<0.001 *vs*. INS1E control. n.s., not significant.

At 11 mM of glucose, hIAPP-INS1E cells did not show changes in ER stress genes, such as CHOP, spliced XBP1 (sXBP1) or ATF3, when compared to rIAPP-INS1E or INS1E control cells (Figure 2A). When cells were exposed to 0.5 µM thapsigargin for 8 hours, the expression of CHOP and ATF3 was significantly higher in hIAPP-INS1E when compared to rIAPP-INS1E or INS1E control cells (Figure 2B). In order to investigate the effect of physiological ER stress inducers, hIAPP-INS1E, rIAPP-INS1E and INS1E cells were cultured at 25 mM glucose for 8 h. However, hIAPP-INS1E cells did not show changes in protein or gene expression as compared to basal 11 mM glucose (data not shown). Conversely, when hIAPP-INS1E cells were treated with 25 mM of glucose together with 400 µM palmitic acid (PA), ER stress markers such as CHOP, ATF3 and sXBP1 significantly increased mRNA levels (Figure 2C) when compared to other controls. These results indicate that the overexpression of hIAPP is sensed by the ER and triggers the activation of the UPR pathway, making hIAPP-INS1E cells more sensitive to ER stress than rIAPP-INSE or INS1E control.

### CHOP inhibition protects from induced ER stress and apoptosis in hIAPP-expressing INS1E cells

**Figure 3:**
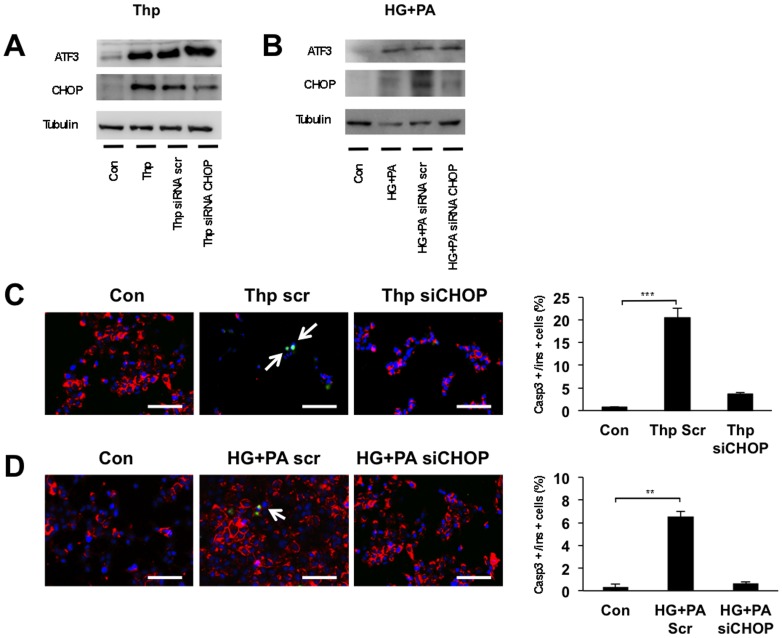
Knockdown of CHOP protects hIAPP-INS1E cells from induced apoptosis. hIAPP-INS1E cells were transfected with 20 µM of siRNA CHOP or siRNA scramble (siRNA scr) as a control. Twenty-four hours after transfection, cells were treated with A) 0.5 µM Thp for 8 hours or B) high glucose (25 mM) and BSA-coupled palmitic acid (400 µM; HG+PA) for 24 hours, and protein expression levels for CHOP, ATF3 and tubulin were determined. Representative Western blotting images are represented from 3 to 5 individual experiments. Immunostaining (left panel) and quantification (right panel) of insulin positive beta-cells (red), cleaved caspase 3 (green) and nuclei (blue) of hIAPP-INS1E cells (Con) and hIAPP-INS1E cells transfected with siRNA scr or siRNA CHOP previously treated with C) Thp or D) HG+PA. Note the absence of cells containing insulin and cleaved caspsase 3 staining in cells transfected with siRNA CHOP as compared to Thp or HG+PA treated controls. Scale bar is 50 µm. Quantification is normalized to number of insulin + cells and expressed as mean ± S.E.M from three independent experiments. **p<0.01 and ***p<0.001 *vs*. controls. No statistical differences were found between controls and Thp siCHOP or HG+PA siCHOP.

We tested whether CHOP was required for the high glucose and palmitic acid-induced ER stress in hIAPP-INS1E cells. Small interfering RNA siCHOP was used to knockdown the expression of CHOP in hIAPP-INS1E cells previously treated for 24 hours with either thapsigargin or high glucose and palmitic acid. Scrambled siRNA was used as a control. As expected, treatment with both thapsigargin or high glucose and palmitic acid induced the expression of CHOP and downstream caspase 3. Upstream ATF3 protein levels were not affected (Figure 3A,B). In hIAPP-expressing cells transfected with siCHOP, thapsigargin or the combination of 25 mM glucose and palmitic acid failed to induce CHOP expression (Figure 3A,B), confirming successful knockdown of CHOP, whereas scrambled siRNA showed similar levels of CHOP when compared to treated controls. Under both conditions, the decrease in CHOP expression was associated with a decrease in the apoptotic marker cleaved caspase 3 (Figure 3C,D), suggesting that the effects observed are CHOP mediated, since the knockdown of CHOP alone is sufficient enough to prevent induced thapsigargin and high glucose and palmitic acid stress and apoptosis.

### Chaperones ameliorate induced ER Stress in INS1E hIAPP cells

**Figure 4:**
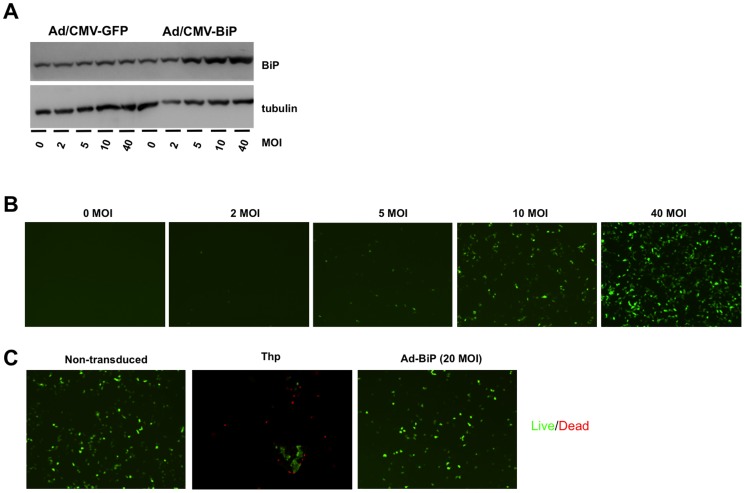
Increased BiP and PDI expression after adenoviral transduction does not affect cell viability. A) hIAPP-INS1E cells were transduced with different doses (MOI) for 24 hours. Representative Western blotting shows BiP protein levels at indicated doses. B) GFP expression after adenoviral (Ad-PDI/GFP) transduction in hIAPP-INS1E cells for 24 hours. Note an increase in GFP expression that correlates with an increase in MOI. C) Live/death viability assay showing no cell death in hIAPP-INS1E cells transduced for 24 hours with Ad-BiP. Western blotting and images in B and C are representative from 3 independent experiments.

**Figure 5:**
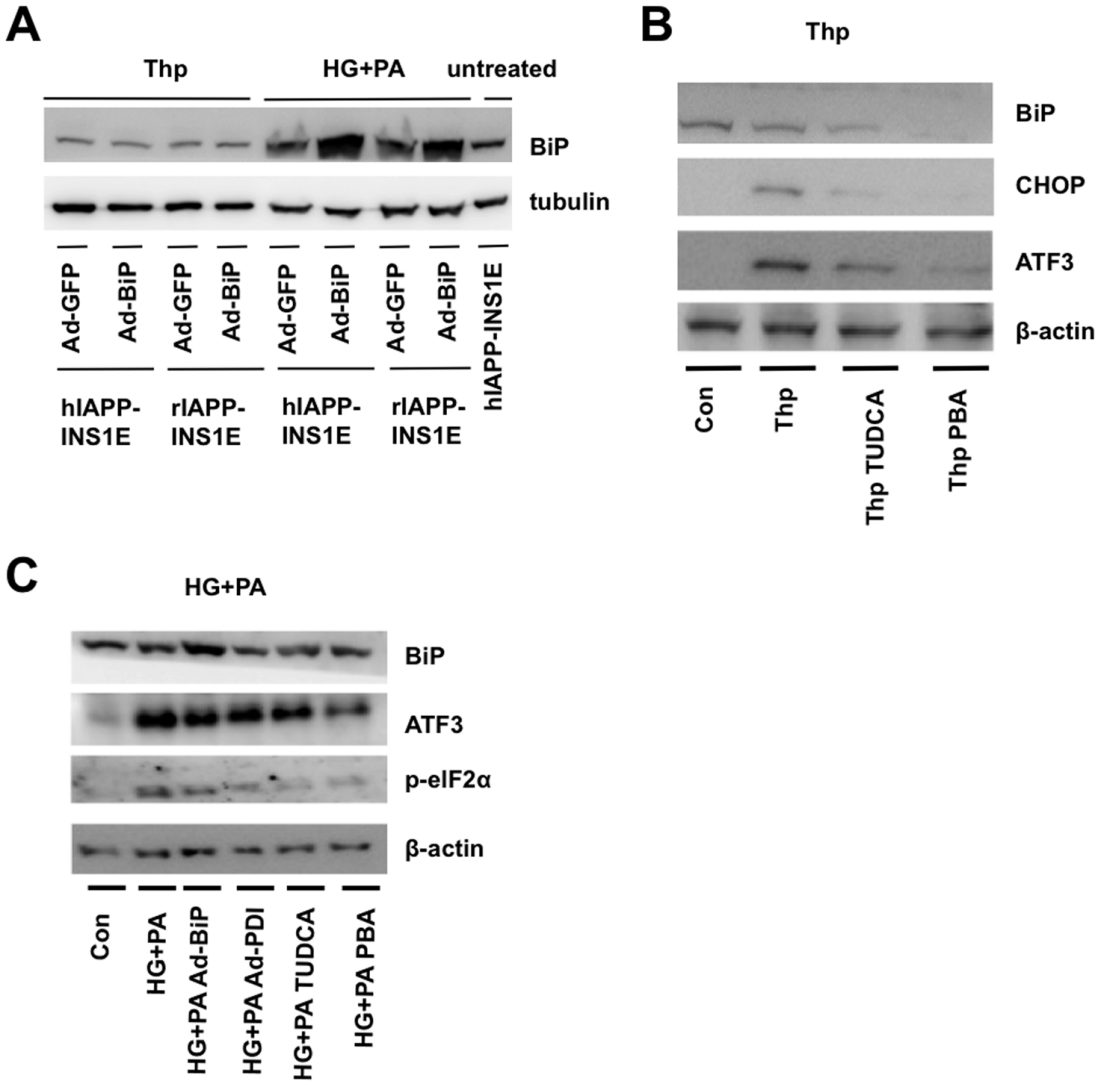
Endogenous and chemical chaperones decrease ER stress markers in hIAPP-INS1E cells. A) BiP and tubulin levels were determined in hIAPP-INS1E cells previously transduced with Ad-BiP or Ad-GFP for 24 hours. Representative western blotting results showing protein expression levels of CHOP, ATF3, BiP, p-eIF2α and β-actin levels in hIAPP-INS1E cells cultured with B) 0.5 µM Thp for 8 hours or C) with 25 mM of glucose and 400 µM palmitic acid (HG+PA) for 24 hours. Representative results from 3 to 5 individual experiments are shown.

In order to increase endogenous chaperone expression, we used adenoviral vectors encoding for the chaperones BiP and PDI. Adenoviral BiP transduction in hIAPP-INS1E showed increased BiP protein expression with increasing MOI of adenovirus (Figure 4A). Adenoviral PDI/GFP expression was similarly tested in hIAPP-INS1E cells (Figure 4B). An MOI of 20 for each adenovirus was chosen for future experiments, based on maximal BiP or PDI/GFP expression in the absence of cell death or detectable cell toxicity (Figure 4C). Although BiP was highly expressed under basal conditions (Figure 4A) and after treatment with high glucose and palmitic acid (Figure 5A), BiP was not overexpressed in hIAPP-INS1E cells in the presence of thapsigargin (Figure 5A), suggesting that thapsigargin blunted adenoviral protein expression.

hIAPP-INS1E cells were cultured in the presence of thapsigargin-containing chemical chaperones TUDCA and PBA for 24 hours. After exposure to thapsigargin, hIAPP-INS1E cells showed high protein expression levels of ER stress markers CHOP and ATF3 (Figure 5B), confirming the mRNA expression results observed in Figure 1B and 1C. When hIAPP-INS1E cells were treated with chemical chaperones TUDCA and PBA, CHOP and ATF3 levels were significantly diminished (Figure 5B), although BiP levels were not recovered. Together, these results suggest that chemical chaperones TUDCA and PBA were able to reduce the hIAPP-potentiated ER stress associated with thapsigargin.

To mimic the plausible protective role of chaperones during high-glucose and palmitic acid-induced ER stress, hIAPP-INS1E cells were incubated with 25 mM glucose and palmitic acid and treated with TUDCA, PBA or previously transduced with Ad-BiP or Ad-PDI for 24 hours. hIAPP-INS1E cells exposed to high glucose and palmitic acid showed an increase in ER stress genes such as ATF3, or phospho Eukaryotic Initiation Factor 2 α (p-eIF2α), when compared to untreated controls (Figure 5C). However, hIAPP-INS1E cells treated with chaperones (TUDCA, PBA, BiP or PDI), demonstrated a decrease in ER stress by showing a decrease in ATF3 and p-eIF2α protein levels (Figure 5C). BiP levels were not affected after exposure to high glucose and palmitic acid, except when hIAPP cells were transduced with adenovirus, confirming the results observed in Figure 5A. Together these data suggest that chaperones are able to ameliorate induced-ER stress in hIAPP-expresing beta-cells; thus, improving chaperone capacity could be important in diminishing hIAPP toxicity.

### Chaperone treatment improves beta-cell function by increasing glucose-stimulated insulin secretion

**Figure 6:**
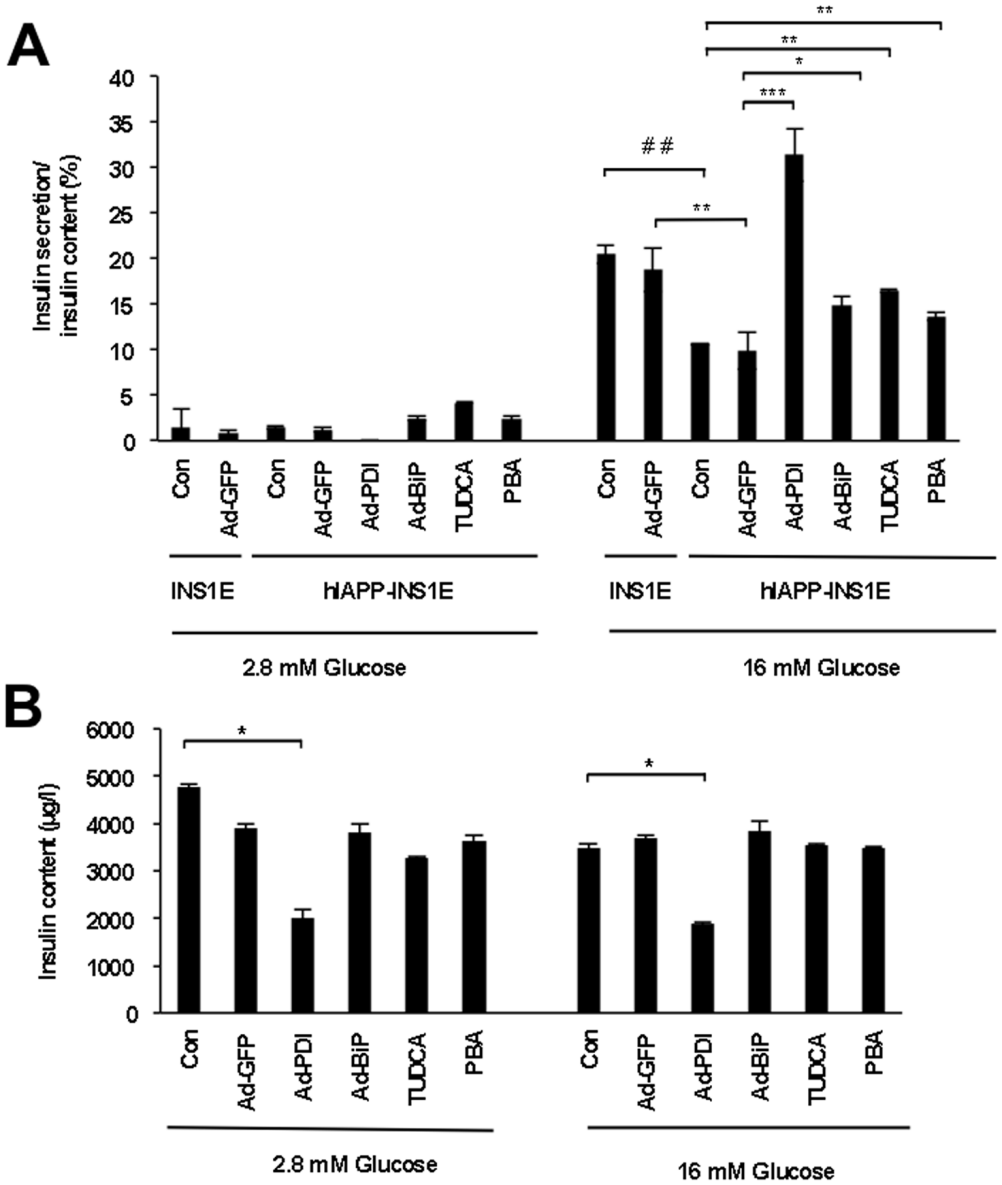
Chaperones ameliorate insulin secretion in hIAPP-INS1E cells. hIAPP-INS1E cells were transduced with Ad-GFP, Ad-BiP, Ad-PDI or treated with TUDCA or PBA for 24 hours. A) Glucose-stimulated insulin secretion was performed at low (2.8 mM) and high (16 mM); glucose expressed as % of insulin content. INS1E control cells were used as a control (Con). B) Total insulin content from same groups was determined in lysates. Results are expressed as mean ± S.E.M from four independent experiments. *p<0.05, **p<0.01, ***p<0.001 vs. hIAPP-INS1E control, *^###^p*<0.001 *vs*. INS1E control. No statistical differences were found in secretion or content between Con and Ad-GFP, BiP, TUDCA and PBA.

To investigate the effect of chaperones on insulin secretion, hIAPP-INS1E cells were either left untreated (Con) or treated for 24 h with Ad-GFP as control, Ad-BiP, Ad-PDI and chemical chaperones TUDCA and PBA. Insulin release from untreated hIAPP-INS1E cells at 16.7 mM glucose was increased in untreated or Ad-GFP treated cells as compared to 2.8 mM glucose. However, overexpression of hIAPP alters glucose-stimulated insulin secretion when compared to INS1E control cells (Figure 6A). Nevertheless, chemical and endogenous chaperone treatment was able to restore and improve glucose-stimulated insulin secretion, although the insulin secretory response of control INS1E cells was not achieved.

The improvement in insulin secretion in hIAPP-INS1E cells treated with BiP, TUDCA and PBA does not appear to be associated with a decrease in insulin content, since all groups had similar levels of insulin in lysates before and after exposure to glucose (Figure 6B). However, PDI treatment of hIAPP-INS1E cells seems to have a detrimental effect on insulin content, suggesting that PDI may be increasing insulin secretion by degranulation of insulin vesicles (Figure 6B).

### Chaperone treatment improves impaired beta-cell function as a result of high glucose and palmitic acid

**Figure 7:**
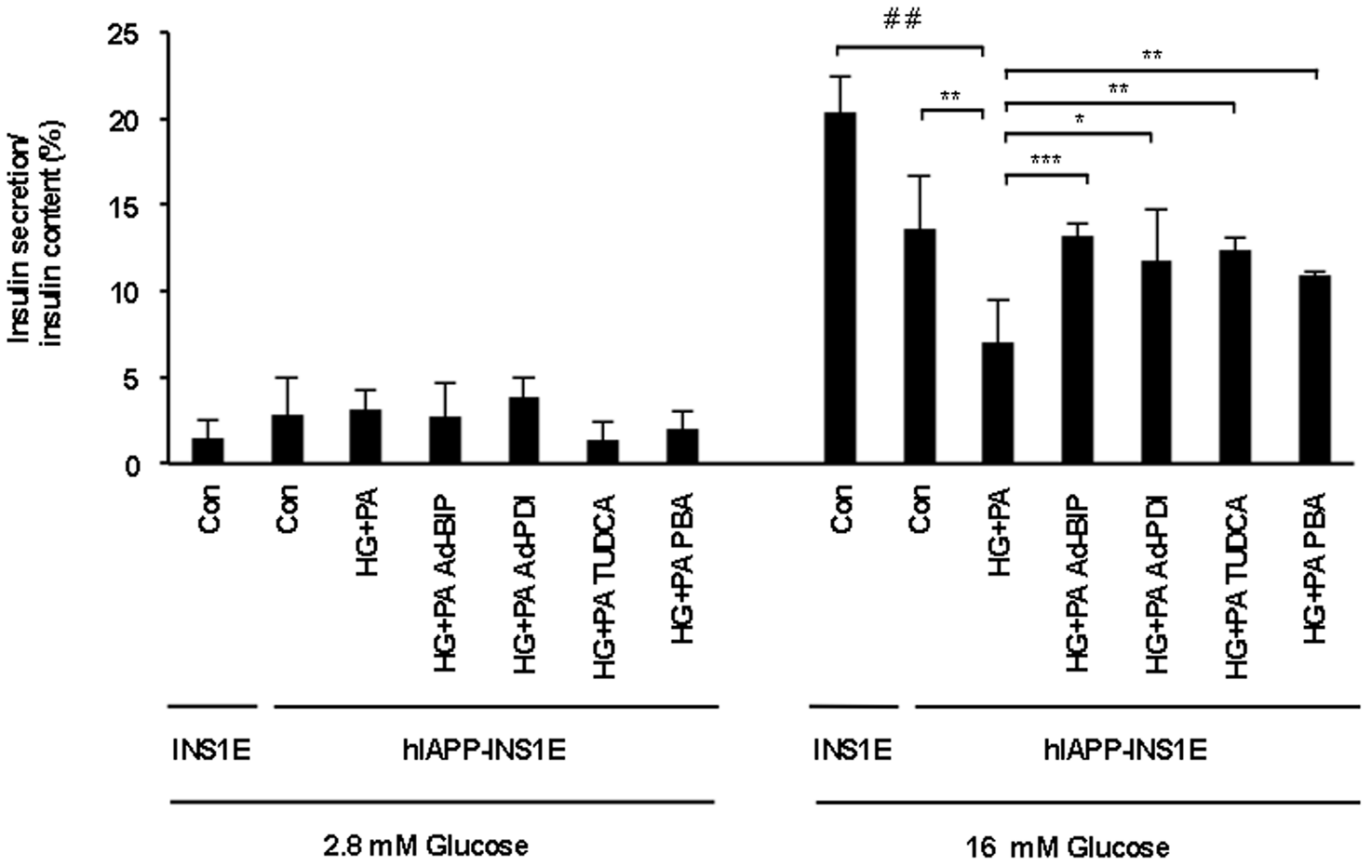
Chaperone treatment prevents beta-cell dysfunction under high glucose and palmitic acid treatment. hIAPP-INS1E cells were transduced with Ad-BiP, Ad-PDI or treated with TUDCA or PBA for 24 hours. After 24 hours, cells were treated with 25 mM of glucose and palmitic acid (HG+PA). Glucose-stimulated insulin secretion was performed at low (2.8 mM) and high (16 mM) glucose using INS1E cells as control as expressed by % of insulin content. Insulin levels were determined by ELISA. Results are expressed as mean ± S.E.M from three independent experiments. ^##^ *p*<0.05 *vs* INS1E control, *p<0.05, **p<0.01, ***p<0.001 *vs*. hIAPP-INS1E cells treated with HG+PA. No statistical differences were found between Con and BiP, PDI, TUDCA and PBA.

In order to study the role of chaperones in insulin release under physiological stress conditions, hIAPP-expressing INS1E were exposed to high glucose and palmitic acid for 24 hours in the presence of chaperones. High glucose and palmitic acid exposure diminished glucose-stimulated insulin release from hIAPP-INS1E cells at 16.7 mM glucose when compared to untreated hIAPP-INS1E cells (Figure 7). In contrast, BiP, PDI, TUDCA and PBA treatment was able to prevent beta-cell dysfunction and maintain insulin secretory response in a similar way to untreated cells. Insulin content of hIAPP-INS1E cells stimulated at 16 mM and 2.8 mM glucose was similar in all groups (data not shown). These results demonstrate that improving chaperone capacity can ameliorate beta-cell function under stressful conditions.

## Discussion

The process of islet amyloid deposition has been recognized as a remarkable physiopathological finding involved in the failure of beta-cell function in T2D [2], [3]. In the present study, we use a previously characterized rat beta-cell line overexpressing the hIAPP transgene that showed intracellular oligomers and a strong alteration to glucose-stimulated insulin and IAPP secretion [26]. We show that thapsigargin or the combination of high glucose and palmitic acid treatment potentiated ER-stress markers via CHOP pathway, and altered the secretory capacity of hIAPP-INS1E cells. By improving chaperone capacity, we have been able to recover ER stress markers and counteract the glucose-stimulated insulin secretion of hIAPP-INS1E cells.

We have previously seen that hIAPP-INS1E cells showed no change in cell death and no change in ER stress marker CHOP when compared to rIAPP-INS1E control cells [26]. Here, we found that the upstream pathways involved in ER stress, such as sXBP1 or ATF3, were not affected, confirming that hIAPP overexpression does not lead to ER stress under basal conditions (11 mM glucose). However, the role of hIAPP in ER-stress induction still needs to be elucidated. In accordance with our studies, Hull et al. demonstrated that overexpression of hIAPP transgenic mice was not associated with significant increases in the expression of ER stress markers [28]. In contrast, some reports have shown that rodent overexpression of hIAPP activates ER stress-mediated apoptosis, leading to a reduction in beta-cell mass [29]. These differences can be explained by the different levels of hIAPP expression or the use of different experimental models, such as transgenic mouse, transgenic rat or INS1E stable cell lines.

Previous studies have shown that high glucose and palmitic acid are potent inducers of ER stress [30], [31]. For example, exposure of islets to high glucose concentrations induces a significant increase in apoptosis [32]. Similarly, increased concentrations of saturated fatty acids are also toxic to islets [9], [33]. Saturated fatty acids impair glucose-stimulated insulin secretion and have a toxic effect on β-cells [34], [35], [36]. Importantly, it has been demonstrated that lipotoxicity is exacerbated in the presence of concomitantly elevated glucose levels [35]. Herein, we show that hIAPP-INS1E cells are more sensitive to exogenous ER stress inducers. Thapsigargin or high glucose and palmitic acid treatment significantly potentiated the expression of ER stress markers, such as CHOP, ATF3 or sXBP1, in hIAPP-expressing cells, when compared to rIAPP- or non-expressing controls, suggesting that hIAPP, but not rIAPP, increased sensitivity to ER stress. The potentiated ER stress activation in hIAPP-INS1E cells can be explained due to the increased aggregation capacity of hIAPP or the formation of intracellular oligomers, which may further affect ER stress [26], [37], [38].

We showed that thapsigargin-induced ER stress was associated with persistent protein upregulation of CHOP and ATF3 in hIAPP-INS1E cells. Similarly, high glucose and palmitic acid ER stress induction was associated with p-eIF2α, ATF3 and CHOP. CHOP is a primary mediator of ER stress-induced apoptosis and is activated upon prolonged ER stress signaling. Here, we demonstrated that either thapsigargin or high glucose and palmitic acid-induced ER stress and apoptosis were dependent of CHOP. Several reports showed that treatment with saturated fatty acids cause numerous alterations that can initiate apoptosis by different mechanisms, including reactive oxygen species, mitochondrial dysfunction, generation of ceramide or induction of CHOP and caspase 3 pathway [9], [13], [39], [40]. In accordance with our results, deletion of CHOP has been shown to enhance beta-cell function and mass in several models of beta-cell stress and T2D [41], [42]. More importantly, this change preceded the induction of cleaved caspase 3, which was apparent after treatment of thapsigargin or high glucose and palmitic acid. Overall, these data suggest that CHOP plays a detrimental role in ER stress induction and that CHOP silencing may be a therapeutic approach to modulating beta-cell function and survival in T2D.

In our studies, activation of ER stress markers was reversed after treatment with chaperones. In thapsigargin or high glucose and palmitic acid treatment, the addition of chemical chaperones TUDCA and PBA was able to prevent activation of ER-stress protein markers. Similar effects were observed after transduction with adenovirus encoding for endogenous chaperones BiP and PDI after high glucose and palmitic acid treatment. In accordance with our results, BiP overexpression has been shown to diminish apoptosis by attenuating the induction of CHOP in ER stress [43].

In line with other reports, we noted that beta-cell overexpression of hIAPP shows a failure in insulin secretion in response to glucose stimulation [26], [44]. The results obtained in hIAPP-INS1E cells demonstrate that treatment with chaperones BiP, TUDCA and PBA ameliorate insulin secretory response under basal conditions. In contrast, PDI showed a marked increase in insulin secretion, accompanied with a significant decrease in insulin content. Although PDI is present in human islets [45] and has been shown to play an important role in sulphide bond formation and isomeration or protein degradation [15], [16], [46], its overexpression has been associated with induced ER stress resulting from accumulation of proinsulin in the ER [14], suggesting that PDI overexpression may have a detrimental effect that disrupts normal insulin processing.

Treatment with high glucose and palmitic acid diminished insulin secretion in hIAPP-INS1E cells, confirming a glucolipotoxic effect [47]. Interestingly, chaperone treatment was able to recover glucose-stimulated insulin secretion. Therapeutic interventions that reduce ER stress have been studied in order to provide strategies for treating ER stress-related human diseases such as T2D [9]. BiP has been shown to be elevated in beta-cells of hIAPP-transgenic mouse models [29]. This increase of BiP can be related to the UPR in response to an increased overload of hIAPP. In addition, BiP has direct interaction with amyloidogenic peptides [48] and has been shown to attenuate the formation of amyloid-like aggregates, suppressing the misfolding of hIAPP [23]. Furthermore, transgenic mice overexpressing BiP specifically in beta-cells were protected against the injury of obesity-induced T2D, maintaining beta-cell function and improving glucose homeostasis [12]. In a similar way, BiP overexpression has been shown to improve insulin sensitivity in ob/ob mice [49]. A promising approach is the use of pharmacological agents, such as orally active chemical chaperones, which can stabilize protein conformation, improve ER folding capacity and facilitate the trafficking of mutant proteins [18], [20], [50], [51], [52], [53]. Ozcan et al. have shown that chemical chaperones, such as PBA and TUDCA, reduce ER stress and restore glucose homeostasis in a mouse model of T2D [18]. In this model, the oral chemical chaperone treatment of obese diabetic mice resulted in the normalization of hyperglycemia and restoration of peripheral insulin sensitivity, thus acting as a potential antidiabetic agent [18]. Furthermore, PBA may provide health benefits by ameliorating insulin resistance and pancreatic β-cell dysfunction in obese subjects [53]. The ability of endogenous and chemical chaperones to alleviate ER stress in transgenic and obese mice models strongly supports the ER stress-based mechanistic model of T2D and demonstrates the feasibility of targeting ER function for therapeutic goals.

In conclusion, our study suggests that ER stress plays a causal role in beta-cell dysfunction in a context of hIAPP overexpression. Furthermore, our results suggest that ameliorating chaperone capacity can be of potential interest for preserving beta-cell function in T2D.
